# Voluntary Exercise Promotes Glymphatic Clearance of Amyloid Beta and Reduces the Activation of Astrocytes and Microglia in Aged Mice

**DOI:** 10.3389/fnmol.2017.00144

**Published:** 2017-05-19

**Authors:** Xiao-fei He, Dong-xu Liu, Qun Zhang, Feng-ying Liang, Guang-yan Dai, Jin-sheng Zeng, Zhong Pei, Guang-qing Xu, Yue Lan

**Affiliations:** ^1^Department of Neurology, National Key Clinical Department and Key Discipline of Neurology, Guangdong Key Laboratory for Diagnosis and Treatment of Major Neurological Diseases, The First Affiliated Hospital, Sun Yat-sen UniversityGuangzhou, China; ^2^Department of Rehabilitation Medicine, The First Affiliated Hospital, Sun Yat-sen UniversityGuangzhou, China; ^3^Department of Rehabilitation Medicine, Guangzhou First People’s Hospital, Guangzhou Medical UniversityGuangzhou, China

**Keywords:** wheel running, inflammation, aging, interstitial fluid, paravascular space, spatial memory

## Abstract

Age is characterized by chronic inflammation, leading to synaptic dysfunction and dementia because the clearance of protein waste is reduced. The clearance of proteins depends partly on the permeation of the blood–brain barrier (BBB) or on the exchange of water and soluble contents between the cerebrospinal fluid (CSF) and the interstitial fluid (ISF). A wealth of evidence indicates that physical exercise improves memory and cognition in neurodegenerative diseases during aging, such as Alzheimer’s disease (AD), but the influence of physical training on glymphatic clearance, BBB permeability and neuroinflammation remains unclear. In this study, glymphatic clearance and BBB permeability were evaluated in aged mice using *in vivo* two-photon imaging. The mice performed voluntary wheel running exercise and their water-maze cognition was assessed; the expression of the astrocytic water channel aquaporin 4 (AQP4), astrocyte and microglial activation, and the accumulation of amyloid beta (Aβ) were evaluated with immunofluorescence or an enzyme-linked immunosorbent assay (ELISA); synaptic function was investigated with *Thy1*–green fluorescent protein (GFP) transgenic mice and immunofluorescent staining. Voluntary wheel running significantly improved water-maze cognition in the aged mice, accelerated the efficiency of glymphatic clearance, but which did not affect BBB permeability. The numbers of activated astrocytes and microglia decreased, AQP4 expression increased, and the distribution of astrocytic AQP4 was rearranged. Aβ accumulation decreased, whereas dendrites, dendritic spines and postsynaptic density protein (PSD95) increased. Our study suggests that voluntary wheel running accelerated glymphatic clearance but not BBB permeation, improved astrocytic AQP4 expression and polarization, attenuated the accumulation of amyloid plaques and neuroinflammation, and ultimately protected mice against synaptic dysfunction and a decline in spatial cognition. These data suggest possible mechanisms for exercise-induced neuroprotection in the aging brain.

## Introduction

The aging brain is mainly characterized by marked cognitive decline, which is a great burden upon both society and an individual’s overall health (Lautenschlager et al., 2008). The loss of protein homeostasis, evident as the accumulation of misaggregated proteins, is an important feature of brain aging and neurodegenerative diseases (Eckert et al., 2008; Rijal Upadhaya et al., 2012). The clearance of misfolded proteins, such as amyloid beta (Aβ), plays a critical role in maintaining protein homeostasis (Rolyan et al., 2011). It has recently been demonstrated that the brain lymphatic–glymphatic system plays a critical role in maintaining the homeostasis within the brain (Arbel-Ornath et al., 2013), because this system is responsible for the clearance of toxins, metabolic waste, and excessive fluid from the brain parenchyma (Iliff et al., 2012; Xie et al., 2013). Transportation across the blood–brain barrier (BBB) is another major efflux mechanism mediating this clearance (Deane et al., 2008). However, the functions of the brain lymphatic–glymphatic system and the BBB are impaired in the aging brain (Kress et al., 2014; Erdö et al., 2017), which disrupts the clearance of toxins and metabolic waste from the brain.

In the aging brain, activated microglia and astrocytes change their morphology and functions to those that are associated with the proinflammatory state (Di Benedetto et al., 2017), and are thought to be major contributors to cognitive impairment (Barrientos et al., 2015). Interventions that reduce inflammation can improve cognition (Ownby, 2010). Neuroinflammation has also been linked to the accumulation of misaggregated proteins, such as Aβ (Raha et al., 2017), or the inflammasome complex (Lage et al., 2014). Aquaporin 4 (AQP4) is a major component of the lymphatic–glymphatic system, and is normally expressed in the end-feet of the astrocytes that facilitate paravascular cerebrospinal fluid (CSF)–interstitial fluid (ISF) exchange (Iliff et al., 2012). However, in aged-brain-related astrogliosis, AQP4 relocalizes from the feet to the somata of astrocytes, thus preventing paravascular CSF–ISF exchange (Ren et al., 2017).

Substantial evidence suggests that increased physical activity or an active lifestyle beneficially affects human brain health (Scarmeas et al., 2009), and reduces the pathological plaque load and improves cognition in patients with AD (Maliszewska-Cyna et al., 2016). However, the mechanisms underlying the effect of physical activity on the reduction of plaque remain unclear. How physical activity specifically contributes to improving brain function is also still unknown, and the effects of physical training on brain efflux clearance, including the glymphatic and BBB functions, and Aβ accumulation during aging have not been fully investigated.

In this study, we explored the mechanisms by which voluntary physical running improves spatial memory and cognition in aged mice. First, the effects of voluntary physical running on the function of the glymphatic pathway and interstitial solute fluid clearance in the aged brain were determined, and the accumulation of Aβ was assessed as an index of the efficiency of ISF clearance. The effects of voluntary physical running on microglial activation and reactive astrogliosis were also assessed. *Thy1–green fluorescent protein (GFP)* transgenic mice specifically express GFP in neurons under the control of the neuron-specific *Thy1* gene (Feng et al., 2000), allowing the direct visualization of the neuron structure at the single-cell level *in vivo* (Ragan et al., 2012), including in the dendrites and dendritic spines. Therefore, we determined the effects of voluntary physical running on dendrites and dendritic spines using *Thy1–GFP* transgenic mice. Synaptic function was also detected by immunofluorescently staining postsynaptic density protein (PSDP95).

## Materials and Methods

### Animals

This study was approved by the Animal Research Committee of the First Affiliated Hospital of Sun Yat-sen University (Guangzhou, China; committee’s reference number: [2013]97). All efforts were made to minimize the number and suffering of animals used in this study. Twenty-four male C57BL/6J mice obtained from the Animal Center of Sun Yat-sen University and 12 *Thy1–GFP* transgenic mice obtained from the Model Animal Research Center of Nanjing University (stock number: 003782) were used. All the animals were used at 14–16 months of age, and were housed under a 12:12 h light–dark cycle (light from 07:00 to 19:00), with controlled temperature and humidity, and given food and water *ad libitum*.

### Voluntary Wheel Training

The animals were randomly divided into two groups, the control group and the physical training (running) group, and each group included 12 C57BL/6J and six *Thy1*–*GFP* male mice. In the control group, the mice were housed in polypropylene cages (36 cm L × 20 cm W × 14 cm H). The mice in the training group were housed in polypropylene cages of the same size, with a 16 cm diameter running wheel, which rotated when a mouse climbed onto the wheel voluntarily. The mice were housed under these conditions for 6 weeks (Littlefield et al., 2015).

### Morris Water Maze

The 12 C57BL/6J mice in each group performed the water-maze task, according to the protocol of van Praag et al. (1999) and Akers et al. (2014). The maze consisted of a circular tub (120 cm in diameter, 50 cm in height) with a white circular platform (10 cm). The tub was surrounded by a curtain located about 1 m from the tub wall, which was painted with distinct geometric cues. The water (24 ± 1°C) was made opaque with white tempera paint to conceal the platform. Over five consecutive days, the platform was submerged 1 cm beneath the surface of the water in the center of one quadrant of the pool. Twenty-four mice underwent four trials (up to 60 s each) per day, starting from each of four different locations in the pool. The animals that failed to locate the platform within the allotted 60 s were gently guided to the platform. All the mice remained on the platform for 10 s at the end of each trial. On day 6, the platform was removed and a single 60 s probe trial was conducted. The swim paths were recorded with an overhead video camera and tracked with automated software (San Diego Instruments, San Diego, CA, USA). The swim speed (mm/s), time to reach the platform during water-maze training, number of the times the target area (former platform) was crossed, and time spent in each quadrant were recorded during the probe trial.

### *In Vivo* Two-Photon Imaging of Glymphatic Pathway Clearance

*In vivo* two-photon imaging of glymphatic pathway clearance was performed in 24 C57BL/6J mice, 12 of which (*n* = 6 per group) were used to evaluate the paravascular space (PVS)–ISF exchange. The other mice were used to evaluate interstitial (ISF) drainage.

For two-photon imaging, the mice were anesthetized with chloral hydrate (4.2%, 0.01 ml/g), and placed securely in a stereotaxic device, with the skull level between the bregma and lambda. The stereotaxic coordinates of the right parietal cortex were 2 mm lateral to the midline and 1.7 mm ahead of the lambdoid (Harvey et al., 2012). A thin cranial window (3 mm in diameter; Golshani and Portera-Cailliau, 2008) was constructed for *in vivo* two-photon imaging.

To evaluate the PVS–ISF exchange, fluorescein isothiocyanate (FITC; 70 kDa; Sigma-Aldrich, Darmstadt, Germany) was dissolved in artificial CSF (ACSF) at a concentration of 1%. A microsyringe (BASi, West Lafayette, IN, USA) was inserted into the cisterna magna, and 10 μl of FITC-dextran was injected within a period of 10 min. To visualize the vasculature, 0.2 ml of rhodamine B (RD; 1% in saline; Sigma) was injected intravenously immediately before imaging.

To evaluate ISF drainage, 5 μl of FITC-dextran at a concentration of 1% in ACSF was injected into the cortex area with a 5 μl syringe fitted with a micropipette controlled by an ultramicro pump syringe mounted onto the stereotaxic device (Carare et al., 2008). The syringe was carefully advanced to a depth 100–150 μm beneath the pial surface, where the dye was delivered at 80 nl/min. Rhodamine B (1% in saline; Sigma) was injected intravenously immediately before imaging to visualize the vasculature.

Two-photon imaging was performed with a custom-built two-photon laser scanning microscope (Leica) at a wavelength of 800 nm and a laser scanning system (Coherent, Santa Clara, CA, USA) equipped with a water immersion objective (25×). To monitor the clearance of ovalbumin injected into the brain parenchyma, lateral images of the *xyz* stacks (512 × 512 pixels, 2 μm resolution) were taken up to 300 μm below the cortical surface. The stacks were collected at 5, 15, 30, 45 and 60 min after the injection of the dye.

### Analysis of BBB Permeability

As described previously, RD (1% in saline) was injected intravenously to visualize the brain vasculature while evaluating the PVS–ISF exchange. Fluorescent intravascular dyes were used to detect leakage from the vasculature (Burgess et al., 2014; He et al., 2016). Therefore, we selected the red fluorescence channel for detection and analyzed the total fluorescence intensity in the extravascular compartment (Nhan et al., 2013). Images were collected at 0, 5, 15, 30 and 60 min after the injection of fluorescent dextran, and were shown as both three-dimensional (3D) stacks and *xyz* stacks.

### Histology

For the histological analysis, the dendrites and dendritic spines of 12 *Thy1–GFP* mice (*n* = 6 per group) were detected, and 12 C57BL/6J mice (*n* = 6 per group) were used for other histological analyses (*n* = 6 per group), including enzyme-linked immunosorbent assays (ELISAs) that detected Aβ1–40 and Aβ1–42. The mice were perfused with 50 ml of ice-cold saline, followed by 50 ml of 4% (w/v) paraformaldehyde in phosphate-buffered saline (PBS; pH 7.4). Their brains were removed and incubated overnight in 4% paraformaldehyde, and then dehydrated in 20%–30% sucrose in PBS. Coronal brain slices (40 μm or 10 μm thick) from the right parietal cortex were sectioned with a frozen microtome (Leica) at intervals of 100 μm to produce consecutive frozen sections. For immunofluorescent staining, the sections were boiled in citric acid buffer (pH 6.0) for 5 min in a microwave oven. After the sections were cooled, they were treated with 0.3% Triton X-100 and 10% goat serum for 1 h at room temperature. The sections were then incubated overnight at 4°C with a primary antibody (1:400 anti-ionized calcium-binding adapter molecule 1 (IBA1) antibody, catalog number 019-19741, Wako, Japan; 1:400 rabbit anti-AQP4 antibody, catalog number AQP014AG0140, Peptide, USA; 1:400 mouse anti-glial fibrillary acidic protein (GFAP) antibody, catalog number nG3893, Sigma-Aldrich; 1:400 mouse anti-Aβ1–16, catalog number ABIN2961826, antibodies-online, Germany; 1:200 mouse anti-Aβ1–40, catalog number 805403, BioLegend, USA; or 1:200 mouse anti-Aβ1–42, catalog number SIG-39142, BioLegend), and then with a secondary antibody (1:300 Alexa-Fluor-488-conjugated goat anti-mouse IgG2a antibody, Life Technologies, catalog number A21131; 1:300 Alexa-Fluor-555-conjugated goat anti-rabbit IgG1 antibody, Life Technologies, catalog number A31572) in PBS containing 10% normal goat serum at room temperature for 1 h. The sections were mounted onto slides, embedded with SlowFade^®^ Gold (Invitrogen), and covered with a coverslip. To evaluate the global AQP4 expression levels, the mean AQP4 immunofluorescence intensity was measured within the cortex and hippocampus. The AQP4 polarity in each image, defined as the ratio of low-stringency areas to high-stringency areas, was also calculated according to Wang et al. (2012; Figure 3). The low-stringency threshold defined the overall area of AQP4 immunoreactivity, whereas the high-stringency threshold defined the area of intense AQP4 immunoreactivity localized to the perivascular end feet.

**Figure 1:**
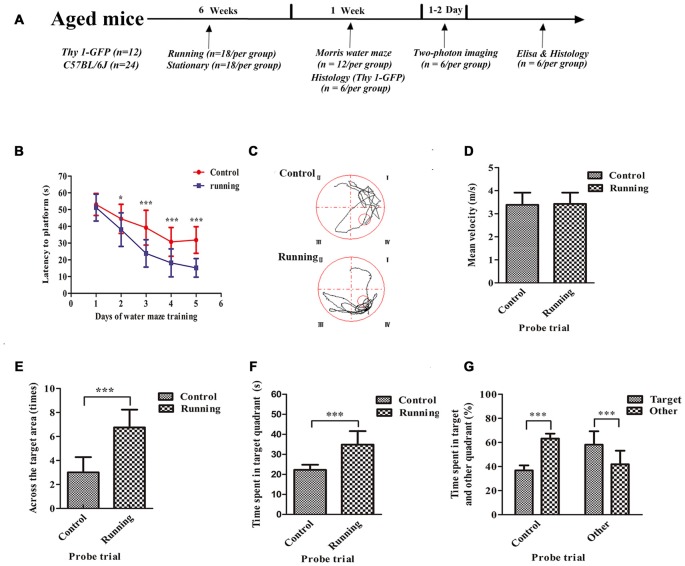
**Schematic diagram of the timeline of this study and the effects of voluntarily running on spatial memory in a water-maze task. (A)** Timeline of the behavioral tests and biochemical parameters used in this study. **(B)** Time to reach the platform in the control and running groups during Morris water-maze training. **(C)** Representative swim paths of mice in the control and running groups during the probe trial. **(D)** Mean velocities of mice in the control and running groups during the probe trial. **(E)** Number of times the target area (former platform) was crossed in the control and running groups during the probe trial. **(F,G)** Time **(F)** and % time **(G)** spent in the target quadrant (formerly contained the platform) in the control and running groups during the probe trial. Datasets are expressed as means ± SD, *n* = 12. *P ≤ 0.05; ***P ≤ 0.001.

**Figure 2:**
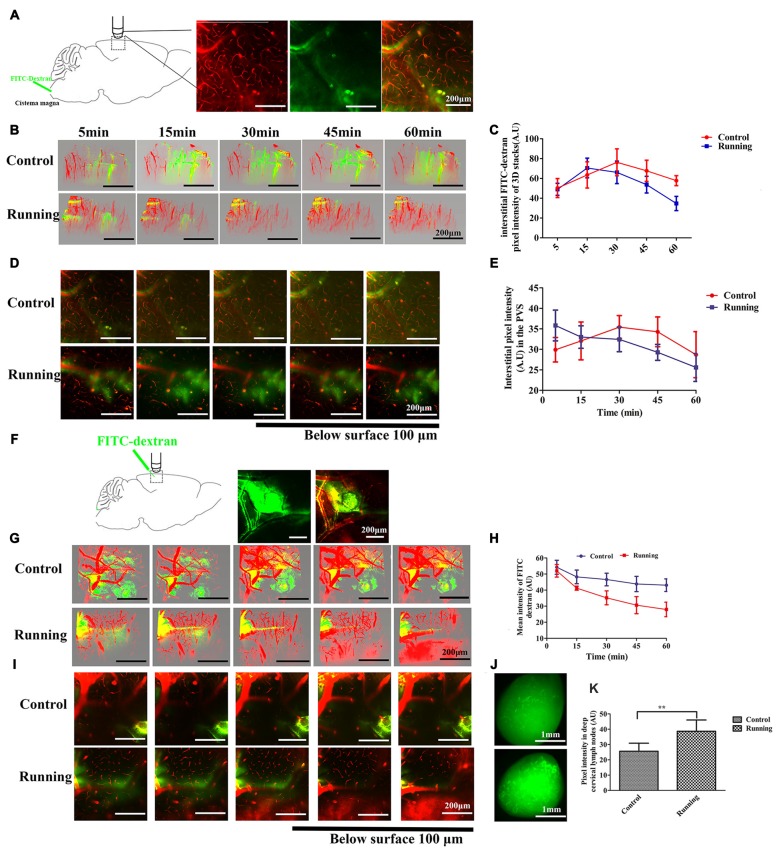
**Analysis of two-photon microscopy data on glymphatic clearance, including the influx through paravascular space (PVS)–interstitial fluid (ISF) exchange and the efflux through ISF drainage. (A)** Schema showing the infusion of the fluorescein isothiocyanate (FITC)-dextran tracer into the cisterna magna for *in vivo* two-photon imaging (250×, scale bar = 200 μm). **(B)** Three-dimensional (3D) images of the brain vasculature and the distribution of the cerebrospinal fluid (CSF) tracer at different time points in the control and running groups (250×, scale bar = 200 μm). **(C)** Quantitative analysis of the mean pixel intensity of the tracer in the 3D image stacks in **(B)**, which shows that the influx and clearance of the CSF tracer were markedly accelerated in the running group compared with the control group (*n* = 6 per group). **(D)** Representative image of the CSF tracer along the perivascular spaces penetrating into the brain parenchyma, 100 μm below the cortical surface (250×, scale bar = 200 μm). **(E)** Quantitative analysis of the fluorescence intensity of the CSF tracer in the PVS shown in **(D)** (*n* = 6 per group). **(F)** Schema showing the dissipation of the FITC-dextran tracer in the brain parenchyma during *in vivo* two-photon imaging, which indicates the efflux of the glymphatic system (100×, scale bar = 200 μm). **(G)** Representative 3D images of dye alignment at different time points in the control and running groups (250×, scale bar = 200 μm). **(H)** Comparison of the average fluorescence intensity in the parenchyma of the control and running groups at different time points (*n* = 6 per group). **(I)** Representative image of the dye alignment along the PVS, 100 μm below the cortical surface (250×, scale bar = 200 μm). **(J)** Representative image of the ISF drainage into the deep cervical lymph nodes in the control and running groups at 1 h after FITC-dextran was injected into the brain parenchyma (50×, scale bar = 1 mm). **(K)** Comparison of the average fluorescence intensity in the deep cervical lymph nodes of the control and running groups (*n* = 6 per group). Datasets are expressed as means ± SD, *n* = 6. **P ≤ 0.01.

**Figure 3:**
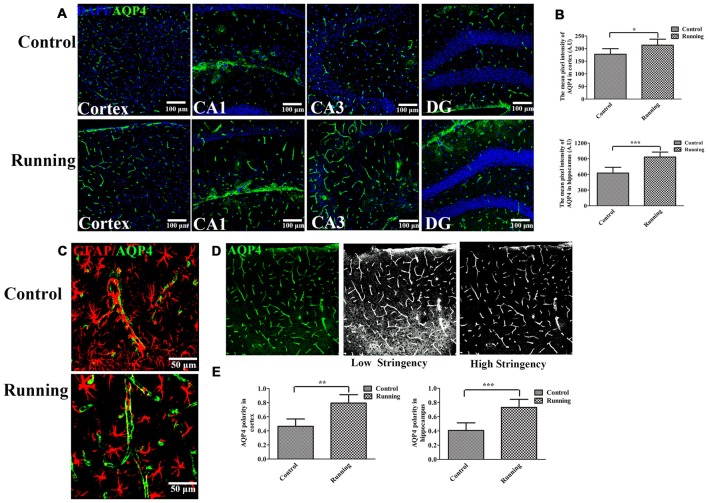
**Effects of voluntarily running on aquaporin 4 (AQP4) expression and AQP4 polarity. (A)** Immunofluorescent staining of AQP4 in the cortex and hippocampus in the control and running groups (250×, scale bar = 100 μm). **(B)** Histograms comparing the average fluorescence intensity of AQP4 in the cortex and hippocampus (*n* = 6 per group). **(C)** Representative images of AQP4 polarity in the control and running groups (250×, zoomed in three-fold, scale bar = 50 μm). **(D)** Schematic diagram of the calculation of AQP4 polarity. **(E)** Histograms comparing AQP4 polarity in the cortex and hippocampus (*n* = 6 per group). Datasets are expressed as means ± SD. *P ≤ 0.05; **P ≤ 0.01; ***P ≤ 0.001.

### ELISAs

Each extracted brain was collected in cold 0.1 M PBS (pH 7.4) and homogenized overnight at −20°C. After two freeze–thaw cycles, the homogenates were centrifuged for 5 min at 5000× *g* at 4°C, and the supernatant was assayed immediately. We determined the levels of Aβ1–40 and Aβ1–42 with ELISA kits (Cusabio Company, Wuhan, China), according to the manufacturer’s instructions. Briefly, serial dilutions of protein standards and the samples were added to 96-well ELISA plates and incubated for 2 h at 37°C. Biotinylated anti-Aβ1–40 or anti-Aβ1–42 antibody (100 μl) was added and the plates were incubated for 1 h at 37°C. After the plates were rinsed with wash buffer, a prepared solution of horseradish-peroxidase- conjugated avidin was added and the plates were incubated for 1 h at 37°C. The substrate solution was added and incubated for 30 min at 37°C. The reaction was stopped with stopping solution. The optical density was detected at 450 nm in a microplate reader (Bio-Rad Laboratories, Hercules, CA, USA). The concentration of each sample was calculated from a linear equation derived from the standard curve constructed from known concentrations of the Aβ peptide.

### Data and Statistical Analyses

The 3D image overlays were visualized with the Leica Application Suite (LAS) Advanced Fluorescence Lite software (LAS AF Lite, 2.4.1 build 6384, Leica). The ImageJ software (National Institutes of Health, Bethesda, MD, USA) was used to analyze the immunohistochemistry results. For the water-maze results and the glymphatic and BBB permeability measurements, two-way repeated measures ANOVA with Sidak’s test for multiple comparisons were performed (Kress et al., 2014). One-way ANOVA and further *LSD-t* test was used to analyze the microglial numbers in the hippocampus. For all other data, an independent-samples *t* test was used. A *P* value <0.05 was considered statistically significant (SPSS 19.0 software, Armonk, NY, USA; Prism 6, GraphPad, La Jolla, CA, USA). Data are expressed as means ± standard deviations of the means (SD).

## Results

### Water Maze

The spatial memories of 24 mice were assessed with the Morris water maze. As shown in Figure 1, two-way ANOVA for repeated measures showed a significant interaction between the group factor and the day of training factor (*F* = 15.19, *P* < 0.001), and the main effects of the group and time factors were significant (*F* = 194.2, *P* < 0.001 and *F* = 208.6, *P* < 0.001, respectively). We used Sidak’s *post hoc* test to analyze the latency periods in detecting the platform during water-maze training. There was no significant difference in the time taken to reach the platform between the control (52.23 ± 6.76 s) and running groups (48.90 ± 10.32 s) on day 1 (*P* > 0.05). However, the time to reach the platform was significantly lower in the running group than in the control group on day 2 (37.99 ± 10.04 vs. 44.23 ± 8.76 s, respectively, *P* < 0.001), day 3 (23.81 ± 8.17 vs. 39.64 ± 10.16 s, respectively; *P* < 0.001), day 4 (18.16 ± 8.35 vs. 30.74 ± 8.54 s, respectively, *P* < 0.001) and day 5 (15.21 ± 5.56 vs. 31.82 ± 7.95 s, respectively, *P* < 0.001; Figures 1B,C). We then evaluated the effect of swimming ability on these times. There was no significant difference in the swimming speeds of the running and control groups (Figure 1D). However, the times taken to cross the target area were significantly increased in the running group (3.00 ± 1.28) than in the control group (6.75 ± 1.49; *t* = 6.628, *P* < 0.001) in the probe trial (Figure 1E). The time spent in the target quadrant was also significantly longer in the running group (34.90 ± 6.73 s, 58.16 ± 11.12%) than in the control group (22.27 ± 2.57 s, 36.83 ± 4.13%; *t* = 6.071, 6.179, *P* < 0.001; Figure 1F). In the control group, the percentage of time spent in the target quadrant (36.83 ± 4.13%) was significantly smaller than the sum of the times spent in the other quadrants (63.17 ± 4.13%, *t* = 15.63, *P* < 0.001). However, in the running group, the percentage of time spent in the target quadrant (58.16 ± 11.12%) was significantly greater than the sum of the times spent in the other quadrants (41.91 ± 11.25%; *t* = 3.542, *P* < 0.01; Figure 1G).

### Glymphatic Clearance

Twelve C57BL/6J mice (*n* = 6 per group) were used to measure the clearance of the paravascular tracer. FITC-dextran was infused into the cisterna magna and the bloodstream was labeled with intravenously injected RD, as shown in Figure 2A. After the intracisternal injection, the FITC tracer moved into the cortex along the penetrating arterioles and entered the interstitium of the parenchyma. Two-way repeated-measures ANOVA indicated a significant main effect of the time factor (*F* = 39.05, *P* < 0.001), and the interaction between the group factor and the time factor was also significant (*F* = 4.786, *P* < 0.01). Sidak’s *post hoc* test was used to evaluate the pixel intensity at different time points within the 3D image stacks (Figures 2B,C). In the control group, the pixel intensity of the FITC-dextran tracer was observed at 5 min (50.322 ± 9.575 arbitrary units [AU]), increased at 15 min (63.523 ± 13.241 AU, *P* < 0.01), and peaked at 30 min (76.293 ± 13.586 AU, *P* < 0.001), after which it decreased slightly at 45 min (67.685 ± 10.724 AU, *P* > 0.05) and 60 min (57.75 ± 5.083 AU, *P* > 0.05). There was no significant difference in the pixel intensity at 5 min and 60 min (*P* > 0.05). In contrast, FITC-dextran was observed at 5 min (48.988 ± 6.049 AU) in the running group, peaked at 15 min (70.510 ± 9.913 AU, *P* < 0.001), decreased slightly at 30 min (66.148 ± 11.372, *P* > 0.05), and then decreased markedly at 45 min (53.692 ± 8.422, *P* < 0.05) and 60 min (34.737 ± 7.17, *P* < 0.001). The pixel intensity was significantly lower at 60 min than at 5 min (*P* < 0.01). There was no significant difference between the running and control groups at 5 min (*P* > 0.05) or 15 min (*P* > 0.05), but the pixel intensities were significantly lower in the running group than in the control group at 30 min (*P* < 0.05), 45 min (*P* < 0.01) and 60 min (*P* < 0.001). We also analyzed the CSF tracer in the perivascular space of the penetrating arteries (Figure 2D), 100 μm below the cortical surface. Two-way repeated-measures ANOVA (Figure 2E) indicated that there was a significant interaction between the group factor and the time factor (*F* = 5.686, *P* < 0.001), and there was a significant main effect of the time factor (*F* = 8.660, *P* < 0.001). The accumulation of the CSF tracer along the perivascular spaces was observed in the control group within 5 min (29.898 ± 2.981 AU), and Sidak’s *post hoc* test showed that the pixel intensity increased at 15 min (32.057 ± 4.618 AU, *P* > 0.05) and peaked at 30 min (35.430 ± 2.811 AU, *P* < 0.05). The fluorescence intensity of the CSF tracer was slightly reduced at 45 min (34.285 ± 3.626, *P* < 0.05) and 60 min (28.705 ± 5.610, *P* > 0.05). In contrast, the pixel intensity along the perivascular spaces in the running group was observed at 5 min (35.823 ± 3.741 AU), but had gradually decreased at 15 min (33.005 ± 2.738 AU, *P* > 0.05), 30 min (32.435 ± 2.999 AU, *P* > 0.05), 45 min (29.277 ± 1.944 AU, *P* < 0.01) and 60 min (25.585 ± 3.412 AU, *P* < 0.001; Figures 2D,E). These results indicate that physical training accelerated the paravascular CSF–ISF exchange in the aging brain.

Twelve C57BL/6J mice (*n* = 6 per group) were used to detect ISF drainage. FITC-dextran was infused into the brain parenchyma as described above, and RD was injected intravenously to label the brain vasculature. As shown in Figure 2F, immediately after the bolus injection, the dye was observed in the parenchyma around the injection site. In the control group, the 3D stacks of images (Figure 2G) showed that the dye diffused into the surrounding parenchyma over a period of 60 min, suggesting the impairment of ISF drainage. However, in the running group, the dye did not diffuse but dissipated along the vessels throughout the 60-min period. Two-way repeated-measures ANOVA indicated a significant interaction between the group factor and the time factor (*F* = 4.973, *P* < 0.01), and significant main effects of the time factor (*F* = 35.02, *P* < 0.001) and group factor (*F* = 49.08, *P* < 0.001). Sidak’s *post hoc* test (Figure 2H) showed that the mean intensity at 5 min did not differ significantly between the the running and control groups (51.99 ± 3.88 AU vs. 54.22 ± 4.32 AU), but was significantly lower in the running group than in the control group at 15 min (41.22 ± 2.77 AU vs. 48.23 ± 4.23 AU, respectively, *P* < 0.05), 30 min (35.17 ± 4.33 AU vs. 46.59 ± 3.95 AU, respectively, *P* < 0.001), 45 min (30.63 ± 5.31 AU vs. 43.81 ± 4.77 AU, respectively, *P* < 0.001) and 60 min (27.95 ± 5.42 AU vs. 43.08 ± 3.92 AU, *P* < 0.001). Our results also indicate that the pixel intensity was not significantly lower at 15 min than at 5 min (*P* > 0.05) in the control group, but was significantly lower at 30 min (*P* < 0.05), 45 min (*P* < 0.01) and 60 min (*P* < 0.01). In contrast, in the running group, the mean intensity of the bolus was significantly lower at 15 min than at 5 min (*P* < 0.01) and this reduction was enhanced at 30 min (*P* < 0.001), 45 min (*P* < 0.001) and 60 min (*P* < 0.001). The images taken 100 μm below the cortical surface (Figure 2I) also showed that the FITC dye dissipated along the paravasculature in the running group, but not in the control group. After the *in vivo* two-photon imaging of ISF drainage, the mice were perfused and the presence of the FITC-dextran dye in the deep cervical lymph nodes was examined (Figure 2J). An independent-samples *t* test showed that the pixel intensity was significantly higher in the running group (38.644 ± 7.368 A.U) than in the control group (25.601 ± 5.266 A.U, *t* = 3.528, *P* < 0.01, Figure 2K), indicating that the ISF drainage into the deep cervical lymph nodes was accelerated in the running group.

### AQP4 Expression and Glial Fibrillary Acidic Protein (GFAP) Localization

The expression of AQP4 was evaluated with laser scanning confocal microscopy, with a 20× objective (Figure 3A). AQP4 expression in the cortex was significantly higher in the running group (213.61 ± 23.70 AU, Figure 3A) than in the control group (177.81 ± 21.78 AU; *t* = 2.72, *P* < 0.05; Figure 3B). We averaged the pixel intensities of the AQP4 deposits in the CA1, dentate gyrus (DG), and CA3 areas of the hippocampus, and the average value was significantly higher in the running group (933.53 ± 95.42 AU) than in the control group (626.92 ± 10.9.90 AU; *t* = 5.16, *P* < 0.001; Figure 3B). We also examined the localization of AQP4 and GFAP expression. As shown in Figure 3C, AQP4 expression is normally polarized insofar as it is expressed within the astrocytic end feet rather than in the astrocytic somata (Raha et al., 2017). In the control group, the immunofluorescence of AQP4 was observed within the astrocytic somata and not in the astrocytic end feet (Figure 3C). However, in the running group, AQP4 was highly polarized and a large proportion of AQP4 immunoreactivity was confined to the perivascular regions (Figure 3C). When the polarity of AQP4 was calculated (Figure 3D), its polarity in the cortex was significantly higher in the running group (11.86 ± 1.95) than in the control group (8.62 ± 1.74; *t* = 3.48, *P* < 0.01; Figure 3E). AQP4 polarity in the hippocampus was also significantly higher in the running group (11.00 ± 1.89) than in the control group (5.91 ± 1.75; *t* = 4.84, *P* < 0.001; Figure 3E).

### BBB Permeability

**Figure 4:**
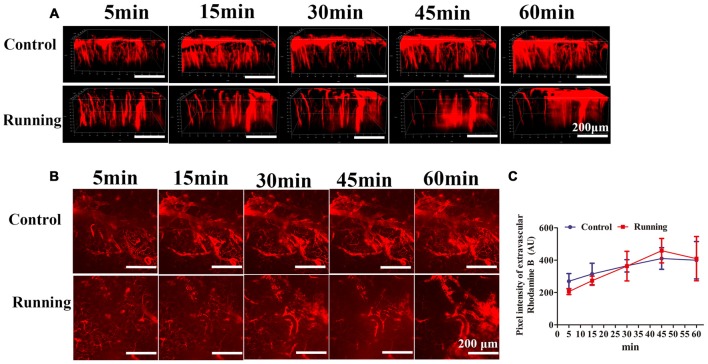
**Effects of voluntarily running on the permeability of the blood–brain barrier (BBB). (A)** 3D reconstructed images of the brain vessels at different time points after rhodamine B was injected into the mouse tail vein. **(B)** *xyz* stacks of the brain vessels at different time points, indicating dye permeation. **(C)** Line diagram of the pixel intensity in the extravascular compartment. Datasets are expressed as means ± SD, *n* = 6.

As shown in Figure 4, the 3D image stacks (Figure 4A) showed that the intravascular dye leaked from the vasculature in both the control and running group. We also analyzed the average fluorescence intensity in the extravascular compartment (Figure 4B). Two-way repeated-measures ANOVA showed that the main effect of time was significant (*F* = 9.472, *P* < 0.001, Figure 4C), but there was no significant interaction effect between group and time (*F* = 1.724, *P* > 0.05). Sidak’s *post hoc* test showed that the average fluorescence intensity in the extravascular compartment in the control group did not differ significantly at 15 min and 30 min (314.667 ± 66.518 AU and 365.167 ± 38.301 AU, respectively; *P* > 0.05) from the pixel intensity at 5 min (269.833 ± 47.275 AU), but was significantly increased at 45 min (410.333 ± 66.818 AU; *P* < 0.01) and 60 min (399.833 ± 115.505 AU; *P* < 0.01). Sidak’s *post hoc* test showed that in the running group, the average fluorescence intensity in the extravascular compartment at 15 min (273.097 ± 28.312 AU) did not differ significantly (*P* > 0.05) from that at 5 min (205.683 ± 18.430 AU), but was significantly increased at 30 min (363.462 ± 91.497 AU; *P* < 0.01), 45 min (458.713 ± 75.439 AU; *P* < 0.001) and 60 min (409.335 ± 136.720 AU; *P* < 0.001). Finally, we compared the average fluorescence intensity in the extravascular compartment at 60 min after RD injection. Turkey’s multiple-comparisons test showed no significant difference between the control and running groups (*P* > 0.05). Our results indicate that physical training had no marked effect on BBB permeability.

### Activation of Astrocytes and Microglia

**Figure 5:**
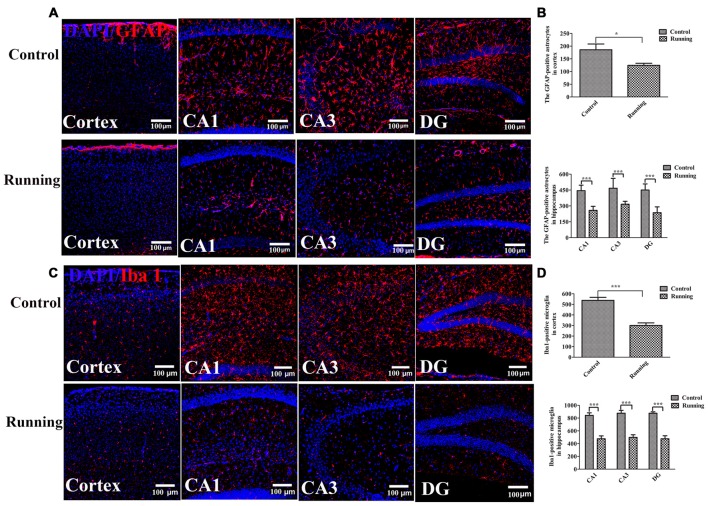
**Effects of voluntarily running on the activation of astrocytes and microglia. (A)** Immunofluorescent staining of glial fibrillary acidic protein (GFAP)-positive astrocytes in the control and running groups (200×, scale bar = 100 μm). **(B)** Histogram comparing the numbers of GFAP-positive astrocytes in the cortex and hippocampus (*n* = 6 per group). **(C)** Immunofluorescent staining of ionized calcium-binding adapter molecule 1 (IBA1)-positive microglia in the control and running groups (200×, scale bar = 100 μm). **(D)** Histogram comparing the numbers of IBA1-positive microglia in the cortex and hippocampus (*n* = 6 per group). Datasets are expressed as means ± SD, *n* = 6. *P ≤ 0.05; ***P ≤ 0.001.

One way ANOVA analysis of the GFAP-positive cells resulted in *F* = 34.840, *P* < 0.001, Further *LSD-t* test indicated the numbers of astrocytes (GFAP-positive cells) in the cortex were significantly lower in the running group (121.17 ± 16.461) than in the control group (185.67 ± 55.773, Figures 5A,B, *P* < 0.05). The numbers of astrocytes in the CA1, DG and CA3 areas were all significantly lower in the running group (258.17 ± 38.128, 316.17 ± 27.716 and 235.50 ± 55.781, respectively) than in the control group (444.00 ± 52.475, 467.00 ± 93.879 and 450.17 ± 57.451, respectively; all *P* < 0.001). One-way ANOVA of IBA1-positive cells resulted in *F* = 34.430, *P* < 0.001. The numbers of microglia (IBA1-positive cells) in the cortex was significantly lower in the running group (300.17 ± 60.088) than in the control group (537.50 ± 70.58, *P* < 0.001; Figures 5C,D), and the numbers of microglia in the CA1, DG and CA3 areas were all significantly lower in the running group (475.50 ± 112.337, 498.00 ± 99.850 and 475.67 ± 111.161, respectively) than in the control group (841.83 ± 98.943, 876.33 ± 107.651 and 876.50 ± 52.755, respectively; all *P* < 0.001).

### Amyloid Plaque Deposition

**Figure 6:**
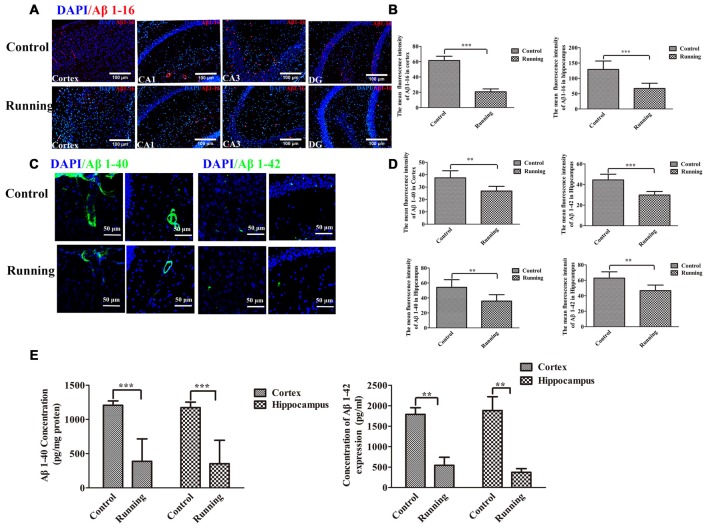
**Effect of voluntarily running on amyloid β accumulation. (A)** Immunofluorescent staining of Aβ1–16 in the control and running groups (200×, scale bar = 100 μm). **(B)** Histograms comparing the average Aβ1–16 fluorescence intensity in the cortex and hippocampus in the control and running groups (*n* = 6 per group). **(C)** Immunofluorescent staining of Aβ1–40 and Aβ1–42 in the control and running groups (250×, zoomed in three-fold, scale bar = 50 μm). **(D)** Histograms comparing the average Aβ1–40 and Aβ1–42 fluorescence intensity in the cortex and hippocampus in the control and running groups (*n* = 6 per group). **(E)** Comparison of the Aβ1–40 and Aβ1–42 levels detected with enzyme-linked immunosorbent assays (ELISAs). Datasets are expressed as means ± SD, *n* = 6. **P ≤ 0.01; ***P ≤ 0.001.

Because the ISF pathway plays a dominant role in Aβ clearance, we assessed the Aβ deposition in the aging mice, as shown in Figure 6. We averaged the pixel intensities of Aβ1–16 deposition in the CA1, DG and CA3 areas of the hippocampus. The mean pixel intensity of Aβ1–16 deposition in the cortex was markedly lower in the running group (20.67 ± 3.72 AU) than in the control group (61.50 ± 5.58 AU; *t* = 14.916, *P* < 0.001; Figures 6A,B), and the mean pixel intensity of Aβ1–16 deposition in the hippocampus (average pixel intensity of the CA1, DG and CA3 areas) was markedly lower in the running group (66.17 ± 16.881 AU) than in the control group (129.00 ± 27.66 AU; *t* = 4.67, *P* < 0.001; Figures 6A,B). We then investigated the accumulation of intraneuronal Aβ1–42 (Eimer and Vassar, 2013) and the deposition of Aβ1–40 outward of the blood vessels (Watts et al., 2014). In the analysis of the pixel intensities of Aβ1–40 and Aβ1–42 (Figures 6C,D), the average fluorescence intensity of Aβ1–40 in the cortex was significantly lower in the running group (26.797 ± 3.809 AU) than in the control group (37.463 ± 5.709 AU; *t* = 3.806, *P* < 0.01), and the average fluorescence intensity of Aβ1–40 in the hippocampus was also significantly lower in the running group (35.802 ± 8.584 AU) than in the control group (54.234 ± 10.104 AU; *t* = 3.405, *P* < 0.01). The average fluorescence intensity of Aβ1–42 in the cortex was significantly lower in the running group (29.813 ± 3.459 AU) than in the control group (44.488 ± 5.509 AU; *t* = 5.986, *P* < 0.001), and the average fluorescence intensity of Aβ1–42 in the hippocampus was also significantly lower in the running group (46.630 ± 7.235 AU) than in the control group (62.839 ± 8.107 AU; *t* = 3.654, *P* < 0.01). We also performed ELISAs of Aβ1–40 and Aβ1–42 to examine their contents. As shown in Figure 6E, the concentrations of Aβ1–40 in the cortex and hippocampus were both significantly lower in the running group (388.61 ± 327.471 pg/mg protein and 353.10 ± 342.004 pg/mg protein, respectively) than in the control group (1204.43 ± 63.398 pg/mg protein and 1173.73 ± 79.421 pg/mg protein, respectively; *t* = 6.016, *P* < 0.001 and *t* = 5.725, *P* < 0.001, respectively). Similarly, the Aβ1–42 content was significantly lower in the cortex (541.772 ± 442.935 pg/mg protein) and hippocampus (371.626 ± 201.174 pg/mg protein) of the running group than in the control group (1789.004 ± 362.113 pg/mg protein and 1882.457 ± 752.716 pg/mg protein, respectively; *t* = 4.875, *P* < 0.01 and *t* = 4.336, *P* < 0.01, respectively).

### Dendrite and Dendritic Spine Loss

**Figure 7:**
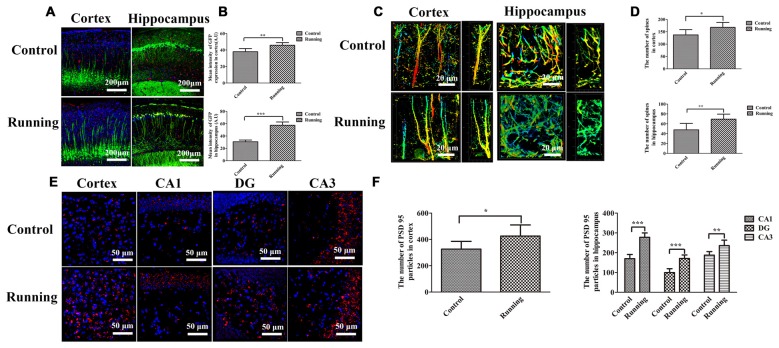
**Effects of voluntarily running on synaptic function detected in *Thy1*–green fluorescent protein (GFP) transgenic mice and on immunofluorescent staining for postsynaptic density protein (PSD95). (A)** Immunofluorescent staining of IBA1-positive cells in the cortices and hippocampi of *Thy1*–GFP mice in the control and running groups (250×, scale bar = 200 μm). **(B)** Histogram comparing the GFP intensity in the cortices and hippocampi of the control and running groups. **(C)** Representative images of dendrites and dendritic spines in the cortices and hippocampus of the control and running groups (630×, zoomed in three-fold, scale bar = 20 μm). To show the spines more clearly, the images were coded with different colors. **(D)** Histogram comparing the number of spines in the cortices and hippocampi of the control and running groups (*n* = 6 per group). **(E)** Immunofluorescent staining of PSD95 in the cortices and hippocampi of the control and running groups (630×, scale bar = 50 μm). **(F)** Histogram comparing the PSD95-positive particles in the cortices and hippocampi of the control and running groups (*n* = 6 per group). Datasets are expressed as means ± SD, *n* = 6. *P ≤ 0.05; **P ≤ 0.01; ***P ≤ 0.001.

*Thy1–GFP* transgenic mice were used to determine the effects of physical training on microglial activation and synaptic plasticity (Figure 7). The lengths of the discrete continuous GFP-positive pixel regions were calculated and averaged to determine a mean arbitrary value for each image, using ImageJ. The mean GFP-positive pixel intensity in the cortex was significantly higher in the running group (45.787 ± 3.189 AU) than in the control group (37.940 ± 3.812 AU; *t* = 3.867, *P* < 0.01; Figures 7A,B), and the mean GFP-positive pixel intensity in the hippocampus was also significantly higher in the running group (57.457 ± 5.340 AU) than in the control group (30.628 ± 2.862 AU; *t* = 10.847, *P* < 0.0001; Figures 7A,B). We also calculated the numbers of dendritic spines in both the cortex and hippocampus (CA1 area; Figure 7C). The numbers of spines in the cortex and hippocampus (CA1 area) were significantly higher in the running group (167.667 ± 20.363 and 69.333 ± 10.270, respectively) than in the control group (137.167 ± 21.198 and 47.667 ± 13.155, respectively; *t* = 2.542, *P* < 0.05 and *t* = 3.180, *P* < 0.01, respectively; Figure 7D). Finally, we calculated the number of PSD95-positive particles (Figure 7E). The number of PSD95-positive particles in the cortex was significantly higher in the running group (425.50 ± 84.88) than in the control group (326.00 ± 59.47; *t* = 2.352, *P* < 0.05; Figure 7F), and the numbers of PSD95-positive particles in the CA1, DG and CA3 areas of the hippocampus were significantly higher in the running group (277.833 ± 22.104, 170.833 ± 17.814 and 235.667 ± 27.998, respectively) than in the control group (169.833 ± 21.414, 99.333 ± 19.694 and 187.500 ± 17.886, respectively; all *P* < 0.001; Figure 7F).

## Discussion

In this study, we examined aging mice to determine the effects of exercise on the disturbance of protein homeostasis and chronic inflammation in the brain, two important aging-related processes. Voluntary running effectively restored the loss of protein homeostasis, seen as a reduction in the accumulation of Aβ deposits. Voluntary running also significantly attenuated the inflammatory activation of microglia and astrocytes. These beneficial effects were accompanied by a significant improvement in glymphatic clearance, but not in BBB function. As a result, cognition was significantly improved in the animals that performed voluntary running. Therefore, this study contributes further evidence of the benefits of exercise to brain health and cognitive function in the elderly.

Inflammation is closely associated with age-related neurodegeneration, including AD (Lucin and Wyss-Coray, 2009), and the inhibition of inflammation can reduce the risk of these diseases (Vlad et al., 2008). Neuroinflammatory glial overreaction and protein accumulation are two prominent features of the aged brain and hallmarks of neurodegenerative diseases, and correlate independently with cognitive dysfunction during aging (Salminen et al., 2011). Many studies suggest that physical exercise might lead to cognitive improvement in normal aging and AD (Huang et al., 2016), however, the confirmatory evidence about the effects of wheel running on neuroinflammation and protein accumulation in aging brain is still lacking. The anti-inflammatory effects of wheel running are well documented in the literatures (Nichol et al., 2008; Kohman et al., 2013). Consistent with those studies, our results show that 6 weeks of training significantly attenuated the activation of microglia and astrocytes (Lee et al., 2015). Furthermore, our data show that the bulk glymphatic flow was accelerated by physical training. Several previous studies have suggested that the transport of protein waste across the BBB is essential for controlling brain homeostasis (Zlokovic et al., 2000; Deane et al., 2008). Furthermore, the dysfunction of the BBB that is associated with inflammation during aging leads to neuronal injury and neurodegeneration (Zhao et al., 2015). It has also been suggested that physical training improves the structural components of the BBB in diabetic rats (de Senna et al., 2015), whereas the effects of physical training on BBB dysfunction in the aging brain have not previously been explored. Here, we used *in vivo* two-photon imaging to study the effects of physical training on BBB function, and found that 6 weeks of voluntary running had no significant effect on BBB permeation. A limitation of our study was that only one training method was tested, so other training programs will be used to investigate the effects of physical training on BBB clearance in a future study. All in all, we have demonstrated that glymphatic clearance was accelerated (although BBB dysfunction was not improved), which reduced the accumulation of Aβ peptides and protected the mice against neurological dysfunction.

The paravascular bulk flow of the CSF and ISF is supported by the astroglial water channel AQP4 (Kress et al., 2014), which localizes to the perivascular astrocytic end feet ensheathing the cerebral vasculature. The genetic deletion of *AQP4* markedly impaired interstitial solute clearance (Iliff et al., 2012). With neuroinflammation and the astrogliotic loss of perivascular AQP4 polarization in the aged brain, the efficiency of CSF–ISF exchange is impaired. However, the role of astrocytic AQP in the aged brain remains controversial. AQP4 is normally predominantly located in the astrocyte foot processes at the borders between major water compartments and the brain parenchyma. Astrocyte dysfunction, such as reactive astrogliosis, disturbs the expression and distribution of AQP4, which in turn, blocks the clearance of brain waste (Heppner et al., 2015). In the present study, reactive astrogliosis was evident in the aged mouse brains. As a result, AQP4 polarization was lost and AQP4 was redistributed from the foot processes to the cell body. AQP4 expression was also significantly reduced in the perivascular end feet immediately surrounding the perforating arteries in the aged brain. Consequently, ISF drainage was impaired.

The accumulation of Aβ peptides in the brain parenchyma is one of the important factors causing neuroinflammation and cognitive decline. The reduced clearance of Aβ has been linked to the deposition of Aβ in the aging brain. Recently accumulating evidence suggests that the brain glymphatic system plays an important role in the clearance of Aβ. For example, it has been reported that the brain glymphatic system is responsible for 40% of the total Aβ clearance during sleep (Xie et al., 2013). We demonstrated that 6 weeks of physical training accelerated Aβ clearance and reduced the accumulation of Aβ peptides in the brain parenchyma by accelerating the movement of ISF drainage fluids. Aβ1–42 and Aβ1–40 have been implicated as the pathogenic and major forms of the Aβ peptide, respectively (Moore et al., 2012), but in the aging brain, there are many other forms of Aβ that contribute to Aβ accumulation (Younkin, 1998; Saito et al., 2011). Therefore, we used Aβ1–16 (6E10) to evaluates the total levels of Aβ (Funamoto et al., 2004), which is considered the best option when studying the aging brain. All these peptides decreased in the running group, which is one of the beneficial effects of physical training that contributes to an improvement in cognition. Thus, the present study provides important new evidence supporting the benefits for AD of exercise-based interventions.

Another factor may be involved in brain synaptic plasticity. Microglia are reported to contribute in various ways to structural synaptic plasticity. They are involved in synaptic pruning, which sustains normal neurological functions during development (Tremblay et al., 2010), and also in synaptic destruction after ischemic injury (Wake et al., 2009). However, microglia can also modulate the synaptic function by secreting cytokines, hormones, or growth factors (Wake et al., 2013). A previous study reported that both the dendritic tree and dendritic spine density in the cortex and hippocampus decrease during aging (Monserrat Hernández-Hernández et al., 2016). As well as reduced microglial activation in the cortex and hippocampus, the expression of GFP-labeled pyramidal neurons and their arbors was increased in the running group, suggesting that physical training prevents microglial activation and the loss of synapsis. Even more interestingly, the arbors were aligned in an orderly manner in the cortex of the aged brain, whereas they were chaotic in the hippocampus, suggesting dysfunctional microglia, because microglia are responsible for pruning. Our results also indicate that the hippocampus is more susceptible to damage than the cortex during aging. However, the arbors in both the cortex and hippocampus were aligned in an orderly way in the running group, indicating that physical training improves microglial function. A limitation of our study was that the mice in the control group were housed in common polypropylene cages, whereas the mice in the running group were housed in cages with running wheels (16 cm diameter). This complex object in the animals’ cages may have enriched their environment, so we cannot exclude the possible effect of environmental enrichment, which is neuroprotective (Beauquis et al., 2013; Hu et al., 2013). We will house the control mice in cages with a similar but locked wheel in future studies.

## Author Contributions

XH, DL, FL, GD and QZ performed the experiments. XH and DL drafted the manuscript. GX and ZP conceived and designed the research. ZP, YL, and JZ edited and revised the manuscript. GX, YL, and ZP approved the final version of the manuscript.

## Funding

This work was supported by grants from the National Natural Science Foundation of China (grant numbers: 81371441, 81372109, 81371255, 81572230, 81572224 and 81671102), the National Key Clinical Department, National Key Discipline, Guangdong Provincial Key Laboratory For Diagnosis and Treatment of Major Neurological Disease (2014B030301035), the Science and Technology Planning Project of Guangdong Province, China (grant numbers: 2016A020213003, 2016B040404053, 2013B051000036, 2013B051000018, 2014B020212001, 2014A030304018, 2014B040404053 and 2016B030230002), the National Science and Technology Support Program (grant numbers: 2015BAI07B01), the Science and Technology Planning Project of Guangzhou, China (grant numbers: 2016201604030036 and 201508020080), and the Fundamental Research Funds for the Central Universities (grant number: 15ykjc14b). The funding bodies had no involvement in the experimental design or the interpretation of the results.

## Conflict of Interest Statement

The authors declare that the research was conducted in the absence of any commercial or financial relationships that could be construed as a potential conflict of interest.

## Abbreviations

Aβ: Amyloid beta
AQP4: aquaporin 4
BBB: blood–brain barrier
CSF: cerebrospinal fluid
FITC: fluorescein isothiocyanate
GFAP: glial fibrillary acidic protein
GFP: green fluorescent protein
IBA1: ionized calcium-binding adapter molecule 1
ISF: interstitial fluid
PVS: paravascular space.
